# Sedimentology of plastics: state of the art and future directions

**DOI:** 10.1098/rsta.2023.0033

**Published:** 2025-10-23

**Authors:** Gary Hampson, Catherine Russell, Ian A. Kane, Michael Clare, Matthew Jackson, Sarah Gabbott

**Affiliations:** ^1^ Earth Science and Engineering, Imperial College London, London, UK; ^2^ Geography and Environment, Loughborough University, Loughborough, UK; ^3^ Earth and Environmental Sciences, The University of Manchester, Manchester, UK; ^4^ National Oceanography Centre, Southampton, UK; ^5^ Geography, Geology and Environment, University of Leicester, Leicester, UK

**Keywords:** plastic, microplastic, sediment, fragmentation, degradation, transport, source to sink

## Abstract

Plastic waste is now a ubiquitous sedimentary material, from the polar ice caps to the deep ocean. Determining the physical and chemical properties, transport pathways and accumulation sites of plastic particles is essential to characterize and mitigate the harmful effects of waste plastic on ecosystems and human health. This requires experimental, modelling and observational approaches across disciplines, including sedimentology, hydrology, chemistry, biology, materials science and many others. Plastic pollution is a deeply complex challenge, and this issue compiles how we may view solutions and understandings through a sedimentological lens. We cover a range of environments, from oceans to rivers and urban stormwater ponds, as well as global questions that consider plastic degradation amid climate change. In this preface, we outline key topics of the papers in this issue and the challenges and opportunities associated with each topic. The special issue illustrates the inherent value and strength of sedimentology as a tool to meaningfully advance our understanding of the transport, accumulation and degradation of plastic waste in the environment.

This article is part of the Theo Murphy meeting issue ‘Sedimentology of plastics: state of the art and future directions’.

## 1. Introduction

The first plastic polymer, Parkesine, was developed by Alexander Parkes in Birmingham in the 1860s [1]. Since 1950, plastics have been produced, used and disposed of in huge quantities, such that approximately 9200 Mt of plastic had been generated by 2017 [2]. It is estimated that over 75% of these plastics have accumulated as waste in landfills and the natural environment [2]. Plastics now constitute a significant sedimentary material, forming particles that are transported, break down and accumulate in the natural environment [3,4]. These particles interact with natural sediment grains and organisms, as well as other pollutants, and can act as vectors for ecotoxins [5,6]. Much recent and current research is concerned with transferring our well-established understanding of natural sediments to characterize how plastics are transported in rivers, coastlines and oceans [7–9]; how they break down by abrasion and weathering [10,11]; how they aggregate and disaggregate [12,13]; and why, when and where they are deposited [14–16]. There are substantial differences in physical and chemical properties between plastic particles and natural sediment grains, such as grain roughness, particle complexity, erosion threshold and much else. As the materials age, there are further differences, including plastics transforming in colour, chemical leaching and changes in their properties, such as elasticity, becoming brittle over time. Consequently, an integrated multidisciplinary approach is needed such that we may appropriately transfer and integrate sedimentary geoscience research with contributions from material scientists, chemical engineers and chemists, fluid dynamicists and biologists, with the goal to understand plastic as a sediment.

This theme issue arose from a Royal Society Theo Murphy meeting on *Sedimentology of plastics: state of the art and future directions*, held on 18−19 March 2024 at the University of Aston, Birmingham, UK. The theme issue comprises 11 papers, excluding this preface, that stem from presentations and posters at the meeting. Presentations at the meeting were organized into four thematic sessions, each summarized below.

### (a) Physical properties and transport

The density, shape and surface textures of plastic particles are still relatively poorly understood but are fundamental in governing their transport, fragmentation and abrasion. For example, the definition of microplastics is disputed [17,18] and refers to particle sizes that imply a spherical shape, which is not supported by observations [19,20]. In part, these differences in definitions reflect the use of different methods (e.g. in field sampling of water and sediment, and in microplastic extraction from samples) and instrumental limitations (e.g. visual microscopy, spectroscopy), which can often be attributed to underlying budget and time constraints and access to analytical facilities. Microplastic particle shape influences their settling velocity [21,22], erosion potential [23], infiltration into beds of natural sediment [24] and ingestion by animals [25]. The concept of hydraulic equivalence [26,27] can be used to identify natural sediment grains whose transport behaviour approximates that of plastic particles; particulate organic carbon grains (‘coffee grounds’) have been suggested as proxies in some studies [8,28–30]. Fragmentation of (primary) plastic particles determines the size distribution of (secondary) plastic particles [31], although there are analytical challenges and sampling biases in characterizing the finer fraction of particle size distributions [32–34]. Laboratory experiments are starting to unravel plastic transport processes, but there is a large parameter space to be explored when considering the combined effects of physical and chemical properties of plastic particles, and laboratory results need to be corroborated in the natural environment.

### (b) Chemical properties and degradation

The surface chemistry and weathering characteristics of plastic particles determine their transport, fragmentation, degradation and propensity to form mixtures and aggregates. Accumulations of plastics and natural sediment may form due to flocculation [13,35] and biofouling [36], which can accelerate plastic transport from ocean surface waters to the seabed [12,37,38] and potentially in rivers, estuaries and other sedimentary environments (cf. natural mud aggregates [39,40]). Floccules and similar aggregates can also act as preferential sites for sorption of other contaminants [41,42]. Degradation of plastics is determined by exposure to oxygen, UV light and water, as well as biotic reactions [43,44]. Plastic polymers have different lifetimes in the environment, reflecting their chemical properties, which can change during environmental exposure [43,44]. Polymers can be designed to be biodegraded rapidly by microbes [45] or recycled [46,47], aiding their potential for recycling and sustainability. However, these polymers may contain similar additives to those in hard-to-recycle polymers, such that their ecotoxic potential is not reduced. Degradation may determine the residence time of plastic particles in the environment, although plastic particles can also be buried beneath overlying sediment. The latter plastic particles are potentially subject to diagenetic processes. For example, additives, plastic monomers and sorbed pollutants can be leached from plastic particles buried in landfill [48,49], while nanoplastics and microplastics can be transported via these leachates into groundwater [50]. Alternatively, the removal of plastic particles from light and oxygen exposure may retard their degradation, for example, following transport to the deep sea. The pathways and intensity with which degrading plastic particles act as vectors and carriers of pollutants in the environment are unclear.

### (c) Monitoring the distribution of plastic sediment grains

Plastics have been sampled and monitored in sedimentary environments that are relatively easy to access (e.g. rivers, floodplains, estuaries and beaches) for over two decades. More recent research has focused on sampling and monitoring plastic particle distributions and fluxes in less accessible sedimentary environments, such as deep-sea canyons and the ocean floor. However, there remain differences in sampling methods, even within the same type of sedimentary environment, which can introduce significant discrepancies in results [51]. Standardization and harmonization of sampling, extraction and processing strategies are required for quantification, comparison and long-term monitoring [20,52,53]. These strategies need to incorporate a robust understanding of processes that govern sediment erosion, transport and deposition to representatively sample spatio-temporal variations in plastic particle distributions due to changing hydrodynamic and sediment transport conditions [20]. Sedimentologically informed monitoring has helped to identify the effects on plastic transport of river floods [7], tidal cycles on beaches [54] and sediment gravity flows in the deep ocean [9,55]. More work is needed to integrate insights from physical experiments into monitoring strategies in sedimentary environments, for example, related to microplastic transport and storage in river bedload [30,56] and to the internal dynamics of turbidity currents [57]. Nanoplastics are absent from most monitoring studies because of challenges in imaging and identifying them [32], but they can make up a significant part of the environmental plastic budget [58]. Another key challenge in any monitoring programme relates to the time spans of observation, since sedimentation and erosional processes are episodic and result in depositional hiatuses across all temporal scales [59].

Monitoring is required not only for individual sedimentary environments but also for environments that are linked, to define the transport pathways of plastic particles from their sources to their sinks. Floodplains are likely to act as transient sinks for plastic particles transported through, and out of, adjacent rivers during overbank floods [16,60]. River channel migration erodes adjacent floodplain sediment, reworking plastic particles from it [61]. During floods, plastic particles can be bypassed through and/or flushed from rivers into deltas, estuaries and/or canyons at their mouths [7,62–64].

### (d) Source-to-sink plastic sediment budgets

Frameworks are needed to construct source-to-sink sediment budgets for plastic particles, which integrate sampling and monitoring studies across different sedimentary environments, use geochemical fingerprinting of plastics to constrain the transport pathways between these environments and establish volumes and fluxes of plastic that move between (and are sequestered within) environments [8,65]. Monitoring should be supplemented and informed by data assimilation techniques and numerical modelling studies. Sources of plastic particles include urban areas and wastewater treatment plants [66–68]. Only a small proportion of plastic waste that enters rivers can be accounted for downstream, implying that riverbed sediments and floodplains constitute a major sink of plastic [68–70]. At the coastline, plastic particles are more abundant at river mouths [14,63,71] and estuaries [72,73]. Only a small proportion of plastics entering the ocean is retained in its surface waters (1%; [74]). It appears likely that most of this plastic is either ‘beached’ along shorelines due to the action of waves, tides and nearshore currents [14,71] or is transported to the ocean floor [75] in sediment gravity flows [9,55] and dense, biofouled aggregates (cf. ‘marine snow’; [12,38]). Plastic particles on the deep-ocean floor are further transported by bottom currents driven by thermohaline circulation, which can winnow and concentrate them [15]. Plastic particles with different physical and chemical properties may take different transport pathways, such that they accumulate in different sinks [76] and have different residence times [43,44]. The residence times and preservation of plastic particles also vary with the environmental conditions of the depositional sinks. Plastic particles deposited in wave-dominated shoreline sinks undergo high rates of physical and chemical weathering through exposure to light, water, oxygen and high-energy waves, resulting in short residence times [10] and enhanced fragmentation [77]. In contrast, plastic particles deposited in cold, oxygen-poor lake-bottom and deep-ocean sinks are likely to have long residence times [76], but even then may be remobilized and flushed to deeper water by ephemeral sediment transport events. Constructing plastic-sediment budgets requires measuring and integrating fluxes of plastic particles between the environmental compartments of a source-to-sink system, combined with characterization of plastic-particle residence times in these environmental compartments. Plastic-sediment budgets can enable prediction of where and when plastic polymers, monomers and additives are released into the environment.

## 2. Contents of the theme issue

The 11 papers in this special issue address aspects of the four research themes outlined above. The physical and chemical properties of plastic particles and their effect on plastic particle transport and degradation are the subject of the first three papers. Wu *et al.* [78] used a coupled fragmentation-sedimentation numerical model to investigate the effects of microplastic-particle fragmentation on their incorporation into aggregates that transport buoyant plastics from ocean surface waters to the ocean floor. Alvarez-Barrantes *et al.* [79] used physical experiments to evaluate how the physical and chemical surface properties of microplastic particles are modified by abrasion by soils and sediments during their transport by wind. Similar physical experiments are used by Ockelford *et al.* [80] to quantify whether the presence of microplastic particles in soils changes the roughness of the soil surface in response to wind erosion.

Six papers address the transport and distribution of plastic particles in sedimentary environments. Alvarez-Barrantes *et al.* [81] used an open-source hydro-morphodynamic numerical model to simulate the sedimentation, erosion and transport of microplastic particles and sediment grains in a braided rive to predict dynamic microplastic distributions and fluxes. Haney *et al.* [82] monitored the influence of baseflow and stormflow on the size and types of plastic particles transported by an urban river in the Great Lakes watershed, North America. Corcoran *et al.* [83] sampled sediment in stormwater ponds, which are designed to prevent pollutants in stormwater runoff from entering natural rivers, to determine their effectiveness as transient sinks for microplastics in the Great Lakes watershed. McGoran *et al.* [84] monitored seasonal trends in microplastic abundance in sediment and ingested by biota from the inner part of the Thames estuary, London, UK. Oh *et al.* [85] characterized the spatio-temporal accumulation at shorelines adjacent to river mouths in northeastern Sicily, southern Italy, of macroplastics and other litter flushed out of rivers during flash floods. Their study used aerial imagery and machine learning, ground-truthed by on-site observations. Pierdomenico *et al.* [86] evaluated the controls on the distribution of macroplastics and other litter in submarine canyons near the coast of southern Italy, using multibeam bathymetry data and remotely operated vehicle videos.

The six papers outlined above recognize the source-to-sink context of the sedimentary environments in their study areas. The final two papers provide larger-scale perspectives consistent with source-to-sink sediment budgets and fluxes for plastic waste. Bakir *et al.* [87] used seafloor physical properties, seafloor micro-litter monitoring data over a 5-year period (2017−2021) and marine-litter sources to condition a machine-learning model that predicts microplastic storage potential and distribution on the UK seabed. Model predictions are intended to guide future sampling and monitoring and to develop a risk-exposure map for seafloor habitats and biota. Pinto da Costa [88] synthesizes our current knowledge of the interactions between plastic pollution and climate change to inform effective mitigation and adaptation strategies.

## 3. Wider-reaching social implications

The presence of plastics in the natural environment is an issue of global scientific and public concern [89]. It is widely accepted that ingestion of microplastics and nanoplastics may have a negative effect on the health of marine and freshwater organisms [90] and potentially also on human health [91,92]. Microplastics can concentrate persistent organic and heavy metal pollutants and may be ingested directly or via contamination through trophic levels by a wide range of organisms, some within the food chain that are eventually consumed by humans [90,93]. However, pollutant concentrations and toxicities vary among different polymers, monomers and additives, making some a greater threat to health [94,95]. Macroplastics can pose additional risks to marine and freshwater organisms through ingestion, entanglement or the potential dispersal of invasive species [96,97]. Even if current efforts to reduce plastic use [98–100] are successful and the supply of plastic into the environment is significantly reduced, existing ‘legacy’ plastic will continue to reside there [101]. The longevity and distribution of the more harmful plastic polymers and their additives in the natural environment depend upon the potential of plastic particles for fragmentation and degradation, as well as the transport pathways and sedimentation of these particles.

Research is needed to provide the evidence base for effective policy to reduce plastic waste. Where do the hazards of ecotoxic plastic polymers and their additives present the greatest risk? What needs to be monitored to characterize this risk, and why? Where and how frequently is monitoring required? Monitoring methods need to be fit for purpose, and the sensitivity of measurements to sampling methods needs to be constrained. An understanding of the sedimentology of plastics needs to be embedded in this research.

## Data Availability

This article has no additional data.
